# (Acetato-κ*O*)bis­(4,4′-dimeth­oxy-2,2′-bi­pyridine-κ^2^*N*,*N*′)copper(II) 3,5-bis­(2,4-di­nitro­phen­yl)-4-nitro­pyrazolate monohydrate

**DOI:** 10.1107/S2414314626010552

**Published:** 2026-10-20

**Authors:** Sherin Anto, Indranil Chakraborty, Raphael G. Raptis

**Affiliations:** a Department of Chemistry and Biochemistry Florida International University, Miami, FL 33199 USA; Vienna University of Technology, Austria

**Keywords:** crystal structure, 5-coordinate Cu(II) cation, pyrazolato anion, solvent disorder

## Abstract

The title salt, [Cu(C_2_H_3_O_2_)(C_12_H_12_N_2_O_2_)_2_](C_15_H_6_N_7_O_10_)·H_2_O, comprises a [Cu(MeObpy)_2_(C_2_H_3_O_2_)]^+^ complex cation (where MeObpy = 4,4′-dimeth­oxy-2,2′-bi­pyridine), a non-coordinating pnpz^−^ anion (pnpz^−^ = 3,5-bis­(2,4-di­nitro­phen­yl)-4-nitro-pyrazolate) and one inter­stitial water solvent mol­ecule. The central Cu^II^ atom is five-coordinate, with a coordination environment inter­mediate between square-pyramidal and trigonal-bipyramidal, defined by two chelating 4,4′-dimeth­oxy-2,2′-bi­pyridine ligands and one O atom of a monodentate acetate anion at 1.969 (3) Å. Two Cu—N bond lengths, 1.985 (3) and 2.006 (3) Å, are similar and shorter than the other two Cu—N bond lengths of 2.061 (3) and 2.148 (4) Å. In the crystal, the electron density from a region of disordered solvent mol­ecules (aceto­nitrile and water) was treated with the SQUEEZE routine in *PLATON* [Spek (2015 ▸). *Acta Cryst*. C**71**, 9–18]. Their contribution was not taken into account during refinement.

## Abstract 2

The complex cation, with a coordination environment of the Cu^II^ atom intermediate between square-pyramidal and trigonal-bipyramidal, crystallizes with a bulky pyrazolato anion and partly disordered solvent molecules.

## Graphical abstract

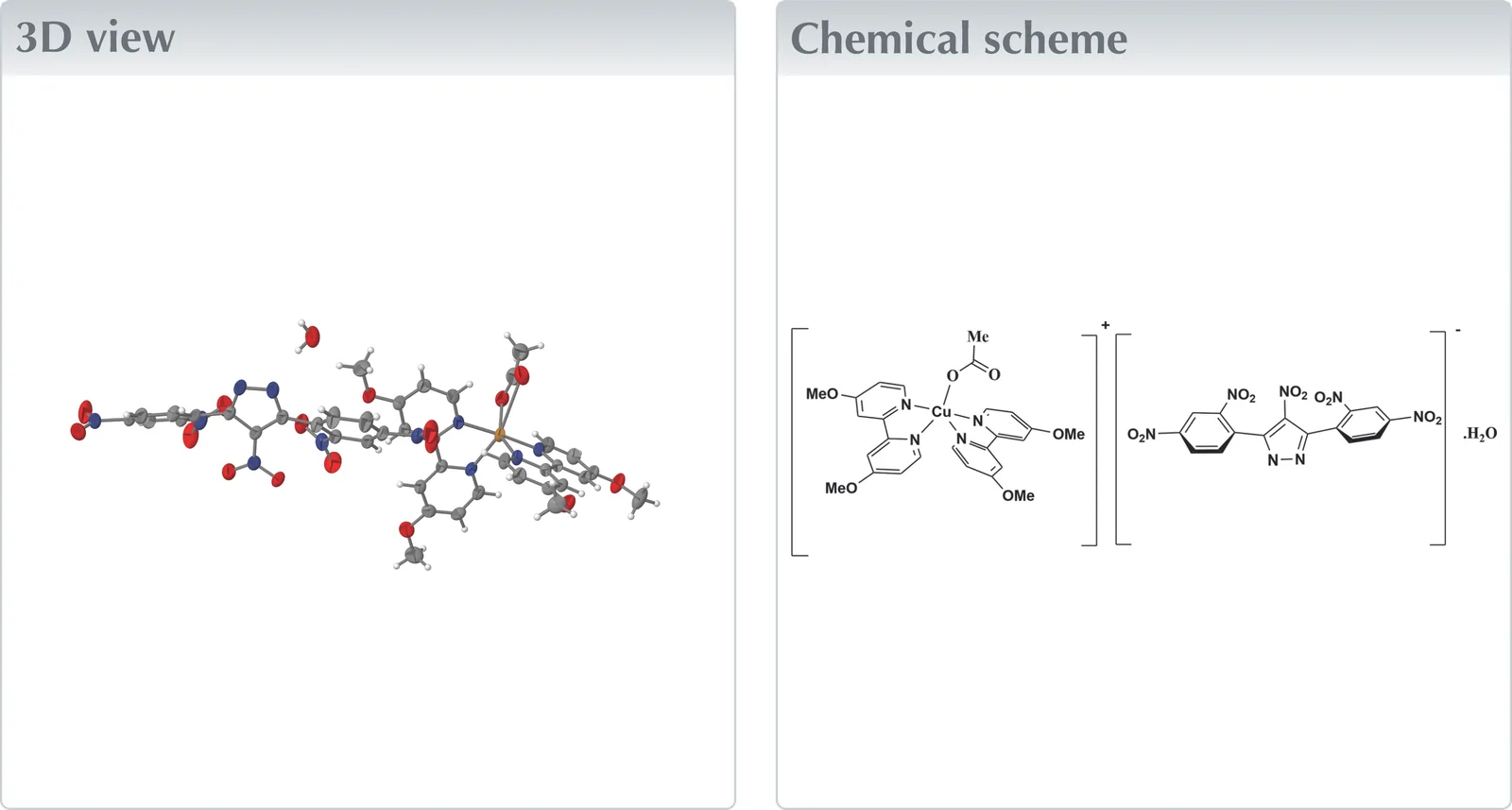

## Structure description

Copper–bi­pyridine complexes have been extensively investigated because of their catalytic properties. Previous studies reported such complexes functioning as catalysts for the hydrolysis of phosphate tri-esters, including those present in highly toxic nerve agents (Morrow & Trogler, 1989 ▸).

The title copper(II) salt, [Cu{4,4′-(MeO)_2_–C_10_H_8_N_2_}(C_2_H_3_O_2_)][3,5-{2,4-(O_2_N)_2_–C_6_H_3_}_2_-4-NO_2_–C_3_N_2_]·H_2_O, containing a dimeth­oxy-substituted chelating bi­pyridine ligand, a non-coordinating pnpz^−^ [pnpz^−^ = 3,5-bis­(2,4-di­nitro­phen­yl)-4-nitro-pyrazolate], and a solvent water mol­ecule, resulted as a product of the reaction with copper hydroxide and dimeth­oxy-substituted bi­pyridine and pnpzH in the presence of base in aceto­nitrile as solvent.

**Figure 1:**
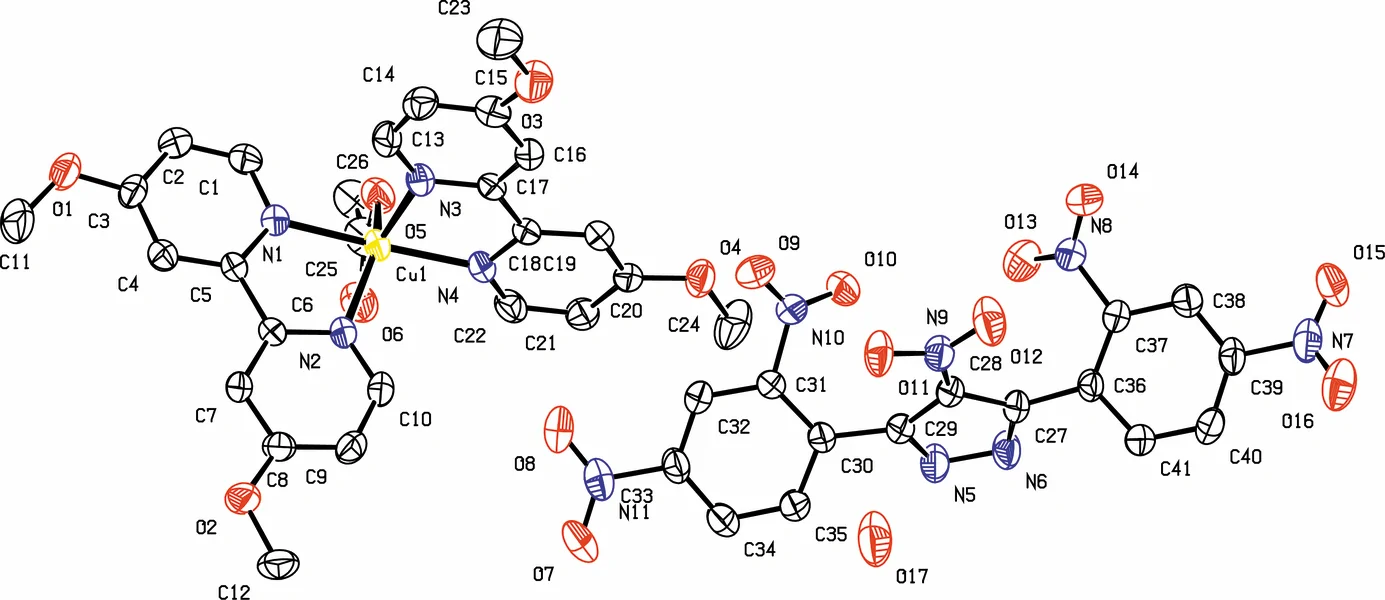
The mol­ecular entities in the structure of the title salt, showing the atom-numbering scheme. Displacement ellipsoids are drawn at the 50% probability level.

The [Cu{4,4′-(MeO)_2_–C_10_H_8_N_2_}(C_2_H_3_O_2_)]^+^ cation (Fig. 1 ▸) exhibits a five-coordinate copper(II) atom with an [N_4_O] coordination set defined by two chelating bi­pyridine ligands and one O atom (O5) of a monodentate acetato ligand. The calculated τ_5_ value (Addison *et al.*, 1984 ▸; Blackman *et al.*, 2020 ▸) is 0.49, indicative of a coordination environment inter­mediate between ideal square-pyramidal (τ_5_ = 0) and trigonal-bipyramidal (τ_5_ = 1), resulting from the elongation of one of the Cu—N bonds [2.148 (4) Å] relative to the others (Table 1 ▸). The Cu1—N1 and Cu1—N4 bond lengths are nearly identical and almost collinear, with an N1—Cu1—N4 angle of 176.15 (12)°. Numerical data for other angles in the coordination sphere is provided in Table 1 ▸. The least-squares plane of the two pyridine rings of each bi­pyridine moiety deviates from co-planarity by 3.38 (19) and 9.04 (19)°. Bond length and angles for this complex mol­ecule are similar to other copper-bi­pyridine derivatives reported elsewhere (Gao *et al.*, 2004 ▸; Hathaway *et al.*, 1980 ▸).

The acetate anion is formed by the hydrolysis of aceto­nitrile solvent in the presence of a base. The dangling oxygen atom (O6) of the acetato ligand is located at a Cu⋯O distance of 2.829 (5) Å. This additional weak metal–acetate inter­action contributes to the distorted coordination environment in the structure (Lobana *et al.*, 2014 ▸). The bite angle of O5—Cu⋯O6 is 50.98 (1)°, which agrees with the reported bite angle for another similar copper-bi­pyridine-acetate complex (Guimaraes *et al.*, 2024 ▸). Within the acetate moiety, the C—O distances of coordinating and non-coordinating O atoms are unsymmetrical (Table 1 ▸), justifying the monodentate classification of acetate coordination in this complex. Similarly, the mean C—O bond length in the carboxyl­ate group, 1.24 (2) Å, also agrees with those reported for other monodentate acetato ligand groups in similar systems [1.25 (2) Å; Hathaway *et al.*, 1980 ▸].

**Figure 2:**
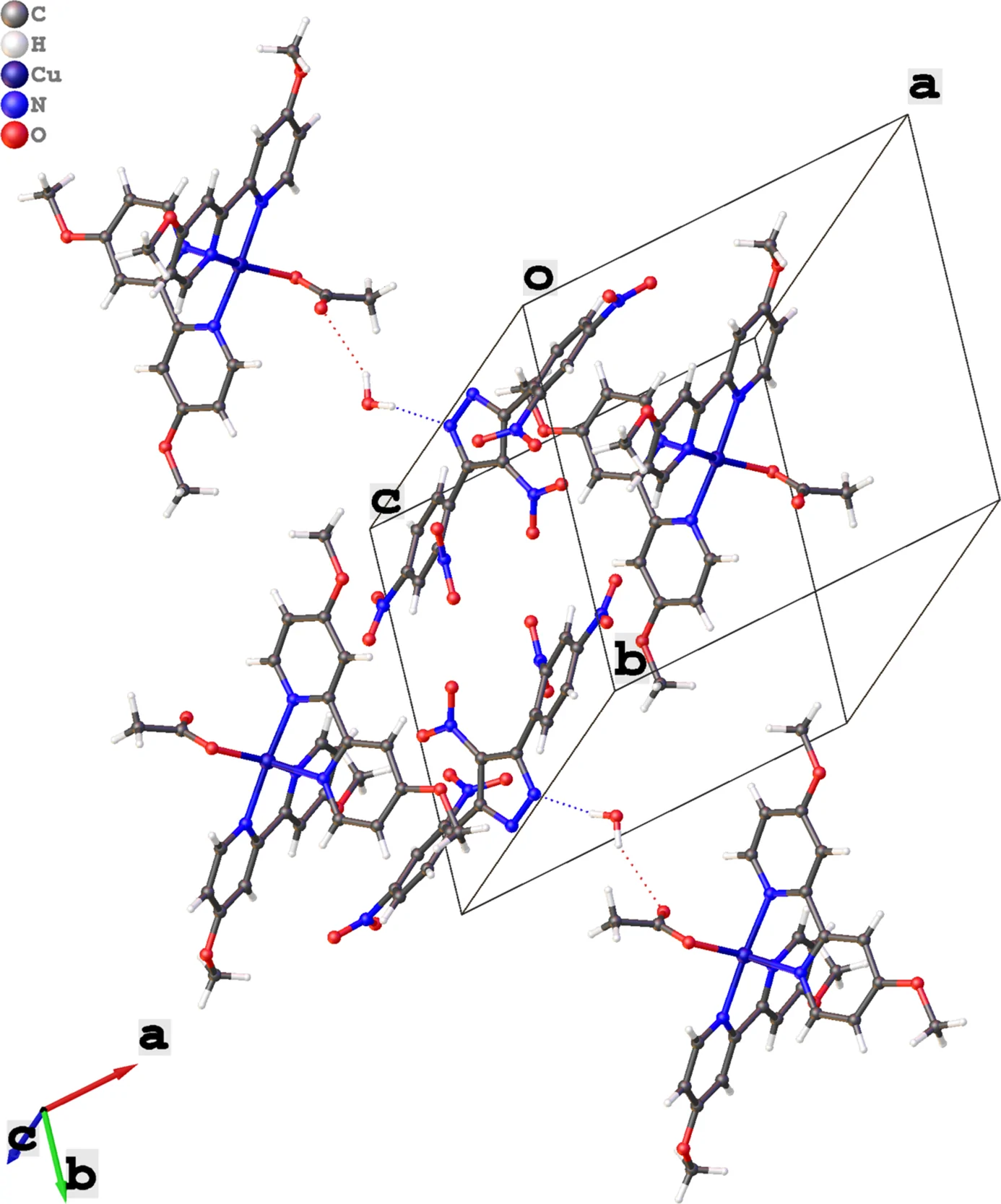
Packing diagram of title complex showing the hydrogen bonds (dotted lines) of the inter­stitial water mol­ecule.

The cationic charge is balanced by the non-coordinating pnpz^−^ anion. Within this anion, the 4-nitro group is approximately co-planar with the five-membered pyrazolato ring [dihedral angle 9.4 (5)°], whereas the four 3,5-nitro groups form dihedral angles between 9.5 (5) and 36.6 (5)° with their corresponding phenyl rings. The presence of five electron-withdrawing nitro groups increases the acidity of the pnpzH ligand, facilitating its deprotonation and stabilizing the Cu^II^ oxidation state (Mathivathanan, 2012 ▸). The inter­stitial solvent water mol­ecule is hydrogen-bonded to the acetato ligand and one of the N atoms of the pyrazolato ring (Table 2 ▸, Fig. 2 ▸).

## Synthesis and crystallization

A 20 ml scintillation vial was charged with Cu(OH)_2_ (25.6 mg, 0.262 mmol), pnpzH (200 mg, 0.398 mmol), 4,4′-dimeth­oxy-2,2′-bi­pyridine (56.7 mg, 0.262 mmol), approximately 0.4 mmol NaOH and 5 ml aceto­nitrile. The mixture was stirred for 24 h at room temperature, filtered through Celite and glass wool to remove unreacted material. The filtrate was set to crystallize by layering of aceto­nitrile with water.

## Refinement

Crystal data, data collection, structure refinement details are summarized in Table 3 ▸. Hydrogen atoms of the water mol­ecule were calculated and included in the model with a fixed O—H distance of 0.85 Å and *U*_iso_(H) = 1.5*U*_eq_(O). Electron density associated with disordered solvent mol­ecules was treated with the SQUEEZE routine in *PLATON* (Spek, 2015 ▸). A solvent mask was calculated and 113 electrons were found in a volume of 433 Å^3^ in one void per unit cell. This is consistent with the presence of one mol­ecule of CH_3_CN and two mol­ecules of H_2_O per asymmetric unit, which account for 104 electrons per unit cell. Their contributions were not taken into account during refinement, and they are not part of the formula and other crystallographic parameters. For treatment of the acetate group, AFIX and RIGU commands (Sheldrick, 2015*b* ▸) were used.

## Supplementary Material

Crystal structure: contains datablock(s) I. DOI: 10.1107/S2414314626010552/wm4263sup1.cif

Structure factors: contains datablock(s) I. DOI: 10.1107/S2414314626010552/wm4263Isup3.hkl

CCDC reference: 2581462

Additional supporting information:  crystallographic information; 3D view; checkCIF report

## Figures and Tables

**Table 1:** Selected geometric parameters (Å, °)

Cu1—O5	1.969 (3)	Cu1—N3	2.148 (4)
Cu1—N4	2.006 (3)	O5—C25	1.260 (6)
Cu1—N1	1.985 (3)	O6—C25	1.229 (5)
Cu1—N2	2.061 (3)		
			
O5—Cu1—N4	91.64 (13)	N4—Cu1—N3	78.42 (15)
O5—Cu1—N1	90.36 (13)	N1—Cu1—N4	176.15 (12)
O5—Cu1—N2	146.88 (12)	N1—Cu1—N2	79.79 (13)
O5—Cu1—N3	111.87 (12)	N1—Cu1—N3	97.78 (15)
N4—Cu1—N2	100.21 (13)	N2—Cu1—N3	100.83 (13)

**Table 2:** Hydrogen-bond geometry (Å, °)

*D*—H⋯*A*	*D*—H	H⋯*A*	*D*⋯*A*	*D*—H⋯*A*
O17—H17*A*⋯N5	0.85	2.08	2.920 (7)	168
O17—H17*B*⋯O6^i^	0.85	2.40	3.168 (7)	151

Symmetry code: (i) 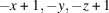.

**Table 3:** Experimental details

Crystal data	
Chemical formula	[Cu(C_2_H_3_O_2_)(C_12_H_12_N_2_O_2_)_2_](C_15_H_6_N_7_O_10_)·H_2_O
*M*_r_	1017.34
Crystal system, space group	Triclinic, *P* 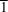
Temperature (K)	293
*a*, *b*, *c* (Å)	13.265 (10), 14.23 (2), 14.302 (14)
α, β, γ (°)	99.87 (4), 102.109 (14), 110.52 (4)
*V* (Å^3^)	2382 (5)
*Z*	2
Radiation type	Mo *K*α
μ (mm^−1^)	0.54
Crystal size (mm)	0.15 × 0.10 × 0.04

Data collection	
Diffractometer	Bruker D8 Quest PHOTON II CCD
Absorption correction	Multi-scan (*SADABS*; Krause *et al.*, 2015 ▸)
*T*_min_, *T*_max_	0.648, 0.745
No. of measured, independent and observed [*I* > 2σ(*I*)] reflections	25044, 8422, 6545
*R*_int_	0.041
(sin θ/λ)_max_ (Å^−1^)	0.599

Refinement	
*R*[*F*^2^ > 2σ(*F*^2^)], *wR*(*F*^2^), *S*	0.054, 0.156, 1.03
No. of reflections	8422
No. of parameters	633
No. of restraints	6
H-atom treatment	H-atom parameters constrained
Δρ_max_, Δρ_min_ (e Å^−3^)	0.97, −1.68

Computer programs: *APEX3* and *SAINT* (Bruker, 2015 ▸), *SHELXT* (Sheldrick, 2015*a* ▸), *SHELXL* (Sheldrick, 2015*b* ▸), *OLEX2* (Dolomanov *et al.*, 2009 ▸) and *publCIF* (Westrip, 2010 ▸).

## full crystallographic data

### (Acetato-κO)bis(4,4'-dimethoxy-2,2'-bipyridine-κ2N,N')copper(II) 3,5-bis(2,4-dinitrophenyl)-4-nitropyrazolate monohydrate Crystal data

**Table:**

[Cu(C_2_H_3_O_2_)(C_12_H_12_N_2_O_2_)_2_](C_15_H_6_N_7_O_10_)·H_2_O	*Z* = 2
*M**_r_* = 1017.34	*F*(000) = 1046
Triclinic, *P*1	*D*_x_ = 1.418 Mg m^−^^3^
*a* = 13.265 (10) Å	Mo *K*α radiation, λ = 0.71073 Å
*b* = 14.23 (2) Å	Cell parameters from 7568 reflections
*c* = 14.302 (14) Å	θ = 2.6–24.8°
α = 99.87 (4)°	µ = 0.54 mm^−^^1^
β = 102.109 (14)°	*T* = 293 K
γ = 110.52 (4)°	Plate, green
*V* = 2382 (5) Å^3^	0.15 × 0.10 × 0.04 mm

### (Acetato-κO)bis(4,4'-dimethoxy-2,2'-bipyridine-κ2N,N')copper(II) 3,5-bis(2,4-dinitrophenyl)-4-nitropyrazolate monohydrate Data collection

**Table:**

Bruker D8 Quest PHOTON II CCD diffractometer	6545 reflections with *I* > 2σ(*I*)
ω–scan	*R*_int_ = 0.041
Absorption correction: multi-scan (SADABS; Krause *et al.*, 2015)	θ_max_ = 25.2°, θ_min_ = 2.5°
*T*_min_ = 0.648, *T*_max_ = 0.745	*h* = −15→15
25044 measured reflections	*k* = −17→17
8422 independent reflections	*l* = −16→17

### (Acetato-κO)bis(4,4'-dimethoxy-2,2'-bipyridine-κ2N,N')copper(II) 3,5-bis(2,4-dinitrophenyl)-4-nitropyrazolate monohydrate Refinement

**Table:**

Refinement on *F*^2^	Primary atom site location: dual
Least-squares matrix: full	Secondary atom site location: diffmap
*R*[*F*^2^ > 2σ(*F*^2^)] = 0.054	Hydrogen site location: mixed
*wR*(*F*^2^) = 0.156	H-atom parameters constrained
*S* = 1.03	*w* = 1/[σ^2^(*F*_o_^2^) + (0.0865*P*)^2^ + 1.8465*P*] where *P* = (*F*_o_^2^ + 2*F*_c_^2^)/3
8422 reflections	(Δ/σ)_max_ = 0.001
633 parameters	Δρ_max_ = 0.97 e Å^−^^3^
6 restraints	Δρ_min_ = −1.68 e Å^−^^3^

### (Acetato-κO)bis(4,4'-dimethoxy-2,2'-bipyridine-κ2N,N')copper(II) 3,5-bis(2,4-dinitrophenyl)-4-nitropyrazolate monohydrate Special details

**Table:**

Geometry. All esds (except the esd in the dihedral angle between two l.s. planes) are estimated using the full covariance matrix. The cell esds are taken into account individually in the estimation of esds in distances, angles and torsion angles; correlations between esds in cell parameters are only used when they are defined by crystal symmetry. An approximate (isotropic) treatment of cell esds is used for estimating esds involving l.s. planes.

### (Acetato-κO)bis(4,4'-dimethoxy-2,2'-bipyridine-κ2N,N')copper(II) 3,5-bis(2,4-dinitrophenyl)-4-nitropyrazolate monohydrate Fractional atomic coordinates and isotropic or equivalent isotropic displacement parameters (Å^2^)

**Table:**

	*x*	*y*	*z*	*U*_iso_*/*U*_eq_	
Cu1	0.51231 (4)	0.50734 (3)	0.74570 (3)	0.03538 (14)	
O2	0.8399 (2)	0.5609 (2)	1.14563 (19)	0.0483 (7)	
O5	0.3525 (2)	0.4402 (2)	0.66754 (18)	0.0476 (6)	
O4	0.6195 (2)	0.2900 (2)	0.39693 (19)	0.0464 (6)	
O11	1.0706 (2)	0.4147 (2)	0.3616 (2)	0.0529 (7)	
N4	0.5560 (2)	0.4303 (2)	0.6410 (2)	0.0352 (6)	
O1	0.4162 (3)	0.7763 (3)	1.0650 (2)	0.0591 (8)	
O10	0.8162 (2)	0.3202 (2)	0.2897 (2)	0.0540 (7)	
O6	0.3461 (3)	0.3151 (3)	0.7414 (2)	0.0598 (7)	
N1	0.4760 (2)	0.5925 (2)	0.8481 (2)	0.0354 (6)	
O3	0.8139 (3)	0.8318 (2)	0.5708 (2)	0.0595 (8)	
N2	0.6330 (2)	0.5219 (2)	0.8709 (2)	0.0356 (7)	
N3	0.6064 (2)	0.6314 (2)	0.6913 (2)	0.0366 (7)	
O9	0.8733 (3)	0.4588 (2)	0.4096 (2)	0.0650 (9)	
O16	1.2430 (3)	0.1411 (2)	−0.1371 (3)	0.0659 (9)	
O12	1.0942 (3)	0.3851 (2)	0.2163 (2)	0.0636 (9)	
N9	1.0564 (3)	0.3548 (2)	0.2820 (2)	0.0408 (7)	
O8	0.7960 (3)	0.3561 (3)	0.7052 (2)	0.0701 (9)	
N10	0.8577 (3)	0.3693 (3)	0.3776 (2)	0.0428 (7)	
O13	0.8539 (3)	0.2405 (3)	0.0662 (3)	0.0669 (9)	
N11	0.8422 (3)	0.2975 (3)	0.6952 (2)	0.0487 (8)	
O7	0.8641 (3)	0.2520 (3)	0.7565 (2)	0.0716 (9)	
N6	0.9334 (3)	0.0778 (2)	0.2054 (2)	0.0458 (8)	
N7	1.1533 (3)	0.1501 (3)	−0.1491 (3)	0.0508 (9)	
N8	0.9287 (3)	0.2699 (3)	0.0280 (2)	0.0487 (8)	
O15	1.0975 (3)	0.1554 (3)	−0.2259 (2)	0.0735 (10)	
N5	0.9046 (3)	0.0960 (2)	0.2913 (2)	0.0471 (8)	
O14	0.9454 (3)	0.3413 (3)	−0.0106 (2)	0.0748 (11)	
C5	0.5387 (3)	0.6175 (3)	0.9426 (2)	0.0298 (7)	
C18	0.6134 (3)	0.4853 (3)	0.5872 (2)	0.0309 (7)	
C6	0.6264 (3)	0.5751 (3)	0.9561 (2)	0.0304 (7)	
C17	0.6489 (3)	0.5991 (3)	0.6201 (2)	0.0317 (7)	
C19	0.6340 (3)	0.4378 (3)	0.5061 (2)	0.0346 (8)	
H19	0.673319	0.477417	0.469892	0.042*	
C20	0.5962 (3)	0.3305 (3)	0.4783 (2)	0.0353 (8)	
C28	0.9955 (3)	0.2479 (3)	0.2669 (2)	0.0344 (7)	
C31	0.8847 (3)	0.3126 (3)	0.4490 (2)	0.0328 (7)	
C4	0.5222 (3)	0.6766 (3)	1.0200 (3)	0.0359 (8)	
H4	0.565120	0.690402	1.085014	0.043*	
C32	0.8629 (3)	0.3375 (3)	0.5380 (3)	0.0353 (8)	
H32	0.841161	0.392278	0.553361	0.042*	
C36	1.0288 (3)	0.1687 (3)	0.1008 (2)	0.0330 (7)	
C27	0.9870 (3)	0.1684 (3)	0.1895 (2)	0.0351 (8)	
C8	0.7756 (3)	0.5448 (3)	1.0537 (3)	0.0374 (8)	
C7	0.6962 (3)	0.5887 (3)	1.0477 (2)	0.0325 (7)	
H7	0.690620	0.626631	1.104799	0.039*	
C29	0.9416 (3)	0.1979 (3)	0.3292 (3)	0.0359 (8)	
C3	0.4397 (3)	0.7152 (3)	0.9983 (3)	0.0401 (8)	
C39	1.1105 (3)	0.1567 (3)	−0.0620 (3)	0.0391 (8)	
C16	0.7187 (3)	0.6663 (3)	0.5808 (3)	0.0369 (8)	
H16	0.747523	0.641418	0.532790	0.044*	
C37	1.0030 (3)	0.2144 (3)	0.0261 (3)	0.0368 (8)	
C25	0.3013 (4)	0.3564 (4)	0.6871 (3)	0.0616 (7)	
C21	0.5383 (3)	0.2747 (3)	0.5339 (3)	0.0402 (8)	
H21	0.512484	0.202531	0.518061	0.048*	
C15	0.7456 (3)	0.7716 (3)	0.6135 (3)	0.0401 (8)	
C13	0.6345 (3)	0.7330 (3)	0.7219 (3)	0.0422 (9)	
H13	0.606787	0.756686	0.771410	0.051*	
C30	0.9205 (3)	0.2335 (3)	0.4241 (2)	0.0363 (8)	
C22	0.5197 (3)	0.3278 (3)	0.6127 (3)	0.0410 (8)	
H22	0.478812	0.289450	0.648802	0.049*	
C33	0.8742 (3)	0.2785 (3)	0.6036 (3)	0.0397 (8)	
C9	0.7837 (3)	0.4920 (3)	0.9675 (3)	0.0422 (9)	
H9	0.836719	0.462977	0.969119	0.051*	
C14	0.7018 (3)	0.8052 (3)	0.6849 (3)	0.0426 (9)	
H14	0.717532	0.875583	0.707684	0.051*	
C38	1.0424 (3)	0.2087 (3)	−0.0563 (3)	0.0410 (8)	
H38	1.022990	0.239400	−0.105722	0.049*	
C41	1.0974 (3)	0.1148 (3)	0.0890 (3)	0.0409 (8)	
H41	1.114949	0.081328	0.136504	0.049*	
C1	0.3944 (3)	0.6280 (3)	0.8280 (3)	0.0437 (9)	
H1	0.349976	0.610154	0.762781	0.052*	
C10	0.7112 (3)	0.4830 (3)	0.8781 (3)	0.0408 (8)	
H10	0.717517	0.447808	0.819980	0.049*	
C2	0.3749 (3)	0.6897 (3)	0.9005 (3)	0.0443 (9)	
H2	0.318851	0.714173	0.884498	0.053*	
C40	1.1395 (3)	0.1100 (3)	0.0093 (3)	0.0430 (9)	
H40	1.186868	0.075552	0.003817	0.052*	
C12	0.9146 (4)	0.5088 (3)	1.1589 (3)	0.0523 (10)	
H12A	0.873123	0.435380	1.128953	0.078*	
H12B	0.948113	0.520619	1.228567	0.078*	
H12C	0.972550	0.535326	1.128253	0.078*	
C34	0.9106 (4)	0.2009 (3)	0.5839 (3)	0.0559 (11)	
H34	0.919952	0.163146	0.629612	0.067*	
C35	0.9332 (4)	0.1794 (4)	0.4950 (3)	0.0580 (12)	
H35	0.957977	0.126343	0.481672	0.070*	
C23	0.8484 (5)	0.9412 (3)	0.6036 (4)	0.0682 (13)	
H23A	0.896324	0.974815	0.566928	0.102*	
H23B	0.888772	0.965131	0.673034	0.102*	
H23C	0.783324	0.957695	0.593137	0.102*	
C24	0.5884 (4)	0.1796 (3)	0.3687 (4)	0.0682 (14)	
H24A	0.611060	0.161408	0.311072	0.102*	
H24B	0.508259	0.144130	0.353733	0.102*	
H24C	0.624991	0.159539	0.422246	0.102*	
C11	0.4734 (5)	0.7986 (4)	1.1676 (3)	0.0735 (15)	
H11A	0.552597	0.836570	1.179257	0.110*	
H11B	0.460869	0.734529	1.186049	0.110*	
H11C	0.445185	0.839579	1.206703	0.110*	
C26	0.1808 (4)	0.3151 (4)	0.6423 (3)	0.0616 (7)	
H26A	0.161544	0.365317	0.614163	0.092*	
H26B	0.143332	0.300361	0.691912	0.092*	
H26C	0.157722	0.252170	0.591003	0.092*	
O17	0.7565 (4)	−0.0713 (3)	0.3540 (3)	0.0811 (11)	
H17A	0.796457	−0.029585	0.327224	0.122*	
H17B	0.754107	−0.130441	0.326910	0.122*	

### (Acetato-κO)bis(4,4'-dimethoxy-2,2'-bipyridine-κ2N,N')copper(II) 3,5-bis(2,4-dinitrophenyl)-4-nitropyrazolate monohydrate Atomic displacement parameters (Å^2^)

**Table:**

	*U*^11^	*U*^22^	*U*^33^	*U*^12^	*U*^13^	*U*^23^
Cu1	0.0423 (3)	0.0443 (3)	0.0240 (2)	0.0214 (2)	0.01217 (18)	0.00847 (18)
O2	0.0553 (16)	0.0564 (17)	0.0345 (14)	0.0343 (14)	0.0002 (12)	0.0054 (12)
O5	0.0496 (14)	0.0608 (13)	0.0327 (13)	0.0249 (10)	0.0086 (11)	0.0113 (10)
O4	0.0613 (17)	0.0394 (15)	0.0438 (15)	0.0242 (13)	0.0238 (13)	0.0057 (12)
O11	0.0668 (19)	0.0408 (15)	0.0436 (15)	0.0155 (13)	0.0251 (14)	−0.0053 (12)
N4	0.0439 (17)	0.0395 (17)	0.0282 (14)	0.0193 (13)	0.0157 (13)	0.0122 (13)
O1	0.073 (2)	0.076 (2)	0.0402 (15)	0.0541 (17)	0.0117 (14)	0.0014 (14)
O10	0.0621 (18)	0.080 (2)	0.0341 (15)	0.0435 (16)	0.0134 (13)	0.0182 (14)
O6	0.0618 (16)	0.0777 (19)	0.0532 (15)	0.0318 (15)	0.0277 (12)	0.0290 (14)
N1	0.0401 (16)	0.0410 (17)	0.0310 (15)	0.0211 (13)	0.0135 (13)	0.0103 (13)
O3	0.080 (2)	0.0362 (15)	0.071 (2)	0.0216 (14)	0.0401 (17)	0.0193 (14)
N2	0.0417 (17)	0.0451 (17)	0.0266 (14)	0.0233 (14)	0.0132 (13)	0.0099 (13)
N3	0.0445 (17)	0.0378 (17)	0.0268 (14)	0.0185 (13)	0.0091 (13)	0.0044 (12)
O9	0.105 (3)	0.0537 (19)	0.067 (2)	0.0545 (18)	0.0367 (19)	0.0280 (16)
O16	0.062 (2)	0.0578 (19)	0.078 (2)	0.0172 (16)	0.0429 (18)	0.0063 (16)
O12	0.090 (2)	0.0438 (17)	0.0542 (18)	0.0136 (15)	0.0421 (17)	0.0099 (14)
N9	0.0457 (18)	0.0398 (18)	0.0381 (17)	0.0179 (14)	0.0159 (14)	0.0069 (14)
O8	0.093 (3)	0.086 (2)	0.0557 (19)	0.053 (2)	0.0426 (18)	0.0147 (17)
N10	0.0520 (19)	0.054 (2)	0.0408 (18)	0.0343 (16)	0.0213 (15)	0.0210 (16)
O13	0.062 (2)	0.089 (2)	0.072 (2)	0.0477 (18)	0.0322 (18)	0.0263 (19)
N11	0.056 (2)	0.051 (2)	0.0333 (17)	0.0154 (17)	0.0178 (15)	0.0045 (15)
O7	0.105 (3)	0.091 (3)	0.0370 (16)	0.048 (2)	0.0317 (17)	0.0298 (17)
N6	0.069 (2)	0.0328 (17)	0.0391 (17)	0.0203 (15)	0.0268 (16)	0.0057 (13)
N7	0.065 (2)	0.0394 (19)	0.052 (2)	0.0178 (16)	0.0361 (19)	0.0076 (16)
N8	0.064 (2)	0.062 (2)	0.0370 (17)	0.0421 (18)	0.0178 (16)	0.0140 (16)
O15	0.121 (3)	0.081 (2)	0.0462 (18)	0.055 (2)	0.046 (2)	0.0282 (17)
N5	0.069 (2)	0.0375 (18)	0.0444 (18)	0.0231 (16)	0.0328 (17)	0.0124 (14)
O14	0.127 (3)	0.089 (3)	0.065 (2)	0.084 (2)	0.051 (2)	0.046 (2)
C5	0.0333 (17)	0.0330 (18)	0.0263 (16)	0.0141 (14)	0.0118 (14)	0.0103 (13)
C18	0.0343 (18)	0.0372 (19)	0.0258 (16)	0.0186 (14)	0.0084 (14)	0.0113 (14)
C6	0.0343 (18)	0.0342 (18)	0.0264 (16)	0.0156 (14)	0.0107 (14)	0.0112 (14)
C17	0.0374 (18)	0.0361 (18)	0.0237 (16)	0.0186 (15)	0.0061 (14)	0.0087 (14)
C19	0.0415 (19)	0.0384 (19)	0.0285 (17)	0.0191 (16)	0.0119 (15)	0.0116 (14)
C20	0.0411 (19)	0.040 (2)	0.0298 (17)	0.0229 (16)	0.0090 (15)	0.0092 (15)
C28	0.043 (2)	0.0316 (18)	0.0309 (18)	0.0183 (15)	0.0121 (15)	0.0056 (14)
C31	0.0403 (19)	0.0343 (18)	0.0285 (17)	0.0186 (15)	0.0112 (14)	0.0104 (14)
C4	0.0381 (19)	0.040 (2)	0.0294 (17)	0.0167 (15)	0.0081 (15)	0.0078 (15)
C32	0.0383 (19)	0.0351 (19)	0.0350 (18)	0.0178 (15)	0.0137 (15)	0.0055 (15)
C36	0.0422 (19)	0.0301 (18)	0.0266 (17)	0.0150 (15)	0.0125 (14)	0.0031 (13)
C27	0.044 (2)	0.0348 (19)	0.0288 (17)	0.0190 (16)	0.0130 (15)	0.0038 (14)
C8	0.042 (2)	0.0352 (19)	0.0349 (19)	0.0181 (16)	0.0068 (16)	0.0103 (15)
C7	0.0397 (19)	0.0326 (18)	0.0270 (17)	0.0174 (15)	0.0109 (14)	0.0049 (14)
C29	0.047 (2)	0.0352 (19)	0.0335 (18)	0.0224 (16)	0.0167 (16)	0.0111 (15)
C3	0.047 (2)	0.041 (2)	0.038 (2)	0.0257 (17)	0.0140 (17)	0.0043 (16)
C39	0.050 (2)	0.0319 (19)	0.0342 (19)	0.0131 (16)	0.0209 (17)	0.0034 (15)
C16	0.045 (2)	0.038 (2)	0.0333 (18)	0.0204 (16)	0.0157 (16)	0.0103 (15)
C37	0.045 (2)	0.038 (2)	0.0324 (18)	0.0222 (16)	0.0143 (16)	0.0063 (15)
C25	0.0649 (15)	0.0672 (13)	0.0534 (15)	0.0251 (11)	0.0167 (12)	0.0216 (12)
C21	0.047 (2)	0.0345 (19)	0.040 (2)	0.0171 (16)	0.0130 (17)	0.0108 (16)
C15	0.041 (2)	0.038 (2)	0.039 (2)	0.0137 (16)	0.0083 (16)	0.0134 (16)
C13	0.054 (2)	0.040 (2)	0.0339 (19)	0.0211 (17)	0.0154 (17)	0.0041 (16)
C30	0.051 (2)	0.0367 (19)	0.0282 (17)	0.0228 (16)	0.0147 (16)	0.0096 (15)
C22	0.051 (2)	0.040 (2)	0.0366 (19)	0.0163 (17)	0.0187 (17)	0.0176 (16)
C33	0.048 (2)	0.044 (2)	0.0260 (17)	0.0184 (17)	0.0122 (16)	0.0050 (15)
C9	0.042 (2)	0.052 (2)	0.042 (2)	0.0300 (18)	0.0130 (17)	0.0092 (17)
C14	0.048 (2)	0.0321 (19)	0.041 (2)	0.0170 (16)	0.0051 (17)	0.0024 (16)
C38	0.055 (2)	0.038 (2)	0.0366 (19)	0.0217 (17)	0.0181 (17)	0.0116 (16)
C41	0.052 (2)	0.044 (2)	0.0324 (19)	0.0273 (18)	0.0119 (17)	0.0090 (16)
C1	0.046 (2)	0.058 (2)	0.0340 (19)	0.0297 (19)	0.0085 (16)	0.0154 (18)
C10	0.048 (2)	0.050 (2)	0.0316 (18)	0.0275 (18)	0.0169 (16)	0.0073 (16)
C2	0.047 (2)	0.054 (2)	0.043 (2)	0.0316 (19)	0.0133 (17)	0.0162 (18)
C40	0.045 (2)	0.045 (2)	0.043 (2)	0.0245 (17)	0.0176 (17)	0.0040 (17)
C12	0.053 (2)	0.055 (3)	0.052 (2)	0.032 (2)	0.0034 (19)	0.017 (2)
C34	0.095 (3)	0.059 (3)	0.038 (2)	0.046 (3)	0.030 (2)	0.024 (2)
C35	0.106 (4)	0.057 (3)	0.046 (2)	0.058 (3)	0.040 (2)	0.027 (2)
C23	0.085 (3)	0.044 (3)	0.084 (4)	0.025 (2)	0.034 (3)	0.028 (2)
C24	0.081 (3)	0.039 (2)	0.083 (3)	0.023 (2)	0.037 (3)	−0.002 (2)
C11	0.095 (4)	0.092 (4)	0.041 (2)	0.059 (3)	0.014 (2)	−0.002 (2)
C26	0.0649 (15)	0.0672 (13)	0.0534 (15)	0.0251 (11)	0.0167 (12)	0.0216 (12)
O17	0.111 (3)	0.071 (2)	0.073 (2)	0.032 (2)	0.061 (2)	0.0169 (19)

### (Acetato-κO)bis(4,4'-dimethoxy-2,2'-bipyridine-κ2N,N')copper(II) 3,5-bis(2,4-dinitrophenyl)-4-nitropyrazolate monohydrate Geometric parameters (Å, º)

**Table:**

Cu1—O5	1.969 (3)	C4—C3	1.390 (5)
Cu1—N4	2.006 (3)	C32—H32	0.9300
Cu1—N1	1.985 (3)	C32—C33	1.381 (5)
Cu1—N2	2.061 (3)	C36—C27	1.488 (5)
Cu1—N3	2.148 (4)	C36—C37	1.387 (5)
Cu1—O6	2.829 (5)	C36—C41	1.397 (5)
O2—C8	1.340 (4)	C8—C7	1.397 (5)
O2—C12	1.433 (5)	C8—C9	1.376 (5)
O5—C25	1.260 (6)	C7—H7	0.9300
O4—C20	1.352 (4)	C29—C30	1.483 (5)
O4—C24	1.435 (5)	C3—C2	1.389 (5)
O11—N9	1.235 (4)	C39—C38	1.360 (5)
N4—C18	1.350 (4)	C39—C40	1.370 (5)
N4—C22	1.321 (5)	C16—H16	0.9300
O1—C3	1.340 (4)	C16—C15	1.381 (6)
O1—C11	1.426 (5)	C37—C38	1.386 (5)
O10—N10	1.229 (4)	C25—C26	1.446 (7)
O6—C25	1.229 (5)	C21—H21	0.9300
N1—C5	1.342 (4)	C21—C22	1.362 (5)
N1—C1	1.347 (5)	C15—C14	1.374 (5)
O3—C15	1.340 (5)	C13—H13	0.9300
O3—C23	1.419 (6)	C13—C14	1.368 (6)
N2—C6	1.355 (4)	C30—C35	1.392 (5)
N2—C10	1.332 (5)	C22—H22	0.9300
N3—C17	1.352 (4)	C33—C34	1.363 (6)
N3—C13	1.326 (5)	C9—H9	0.9300
O9—N10	1.204 (5)	C9—C10	1.384 (5)
O16—N7	1.220 (5)	C14—H14	0.9300
O12—N9	1.226 (4)	C38—H38	0.9300
N9—C28	1.402 (5)	C41—H41	0.9300
O8—N11	1.203 (5)	C41—C40	1.372 (5)
N10—C31	1.468 (5)	C1—H1	0.9300
O13—N8	1.214 (5)	C1—C2	1.368 (5)
N11—O7	1.222 (5)	C10—H10	0.9300
N11—C33	1.469 (5)	C2—H2	0.9300
N6—N5	1.371 (4)	C40—H40	0.9300
N6—C27	1.320 (5)	C12—H12A	0.9600
N7—O15	1.219 (5)	C12—H12B	0.9600
N7—C39	1.475 (5)	C12—H12C	0.9600
N8—O14	1.213 (5)	C34—H34	0.9300
N8—C37	1.466 (5)	C34—C35	1.374 (6)
N5—C29	1.325 (5)	C35—H35	0.9300
C5—C6	1.481 (5)	C23—H23A	0.9600
C5—C4	1.375 (5)	C23—H23B	0.9600
C18—C17	1.476 (5)	C23—H23C	0.9600
C18—C19	1.368 (5)	C24—H24A	0.9600
C6—C7	1.375 (5)	C24—H24B	0.9600
C17—C16	1.367 (5)	C24—H24C	0.9600
C19—H19	0.9300	C11—H11A	0.9600
C19—C20	1.382 (5)	C11—H11B	0.9600
C20—C21	1.379 (5)	C11—H11C	0.9600
C28—C27	1.395 (5)	C26—H26A	0.9600
C28—C29	1.393 (5)	C26—H26B	0.9600
C31—C32	1.380 (5)	C26—H26C	0.9600
C31—C30	1.387 (5)	O17—H17A	0.8500
C4—H4	0.9300	O17—H17B	0.8500
			
O5—Cu1—N4	91.64 (13)	C38—C39—N7	118.2 (3)
O5—Cu1—N1	90.36 (13)	C38—C39—C40	122.4 (3)
O5—Cu1—N2	146.88 (12)	C40—C39—N7	119.3 (3)
O5—Cu1—N3	111.87 (12)	C17—C16—H16	120.5
N4—Cu1—N2	100.21 (13)	C17—C16—C15	118.9 (3)
N4—Cu1—N3	78.42 (15)	C15—C16—H16	120.5
N1—Cu1—N4	176.15 (12)	C36—C37—N8	121.5 (3)
N1—Cu1—N2	79.79 (13)	C38—C37—N8	115.4 (3)
N1—Cu1—N3	97.78 (15)	C38—C37—C36	123.1 (3)
N2—Cu1—N3	100.83 (13)	O5—C25—C26	112.6 (4)
C8—O2—C12	118.6 (3)	O6—C25—O5	124.9 (5)
C25—O5—Cu1	112.1 (3)	O6—C25—C26	122.5 (5)
C20—O4—C24	117.9 (3)	C20—C21—H21	120.8
C18—N4—Cu1	117.7 (2)	C22—C21—C20	118.4 (4)
C22—N4—Cu1	124.1 (2)	C22—C21—H21	120.8
C22—N4—C18	117.6 (3)	O3—C15—C16	115.1 (3)
C3—O1—C11	118.7 (3)	O3—C15—C14	126.2 (4)
C5—N1—Cu1	117.5 (2)	C14—C15—C16	118.8 (3)
C5—N1—C1	118.6 (3)	N3—C13—H13	118.0
C1—N1—Cu1	123.9 (2)	N3—C13—C14	124.0 (3)
C15—O3—C23	118.0 (3)	C14—C13—H13	118.0
C6—N2—Cu1	113.8 (2)	C31—C30—C29	127.2 (3)
C10—N2—Cu1	128.5 (2)	C31—C30—C35	115.8 (3)
C10—N2—C6	117.6 (3)	C35—C30—C29	116.9 (3)
C17—N3—Cu1	113.2 (2)	N4—C22—C21	124.2 (3)
C13—N3—Cu1	129.8 (3)	N4—C22—H22	117.9
C13—N3—C17	116.9 (3)	C21—C22—H22	117.9
O11—N9—C28	118.4 (3)	C32—C33—N11	119.1 (3)
O12—N9—O11	122.6 (3)	C34—C33—N11	119.3 (3)
O12—N9—C28	119.1 (3)	C34—C33—C32	121.5 (3)
O10—N10—C31	117.1 (3)	C8—C9—H9	120.7
O9—N10—O10	124.9 (3)	C8—C9—C10	118.5 (3)
O9—N10—C31	117.9 (3)	C10—C9—H9	120.7
O8—N11—O7	124.0 (4)	C15—C14—H14	120.7
O8—N11—C33	118.5 (3)	C13—C14—C15	118.6 (4)
O7—N11—C33	117.5 (4)	C13—C14—H14	120.7
C27—N6—N5	108.3 (3)	C39—C38—C37	117.5 (3)
O16—N7—C39	116.6 (4)	C39—C38—H38	121.3
O15—N7—O16	125.7 (4)	C37—C38—H38	121.3
O15—N7—C39	117.7 (4)	C36—C41—H41	119.1
O13—N8—C37	118.2 (3)	C40—C41—C36	121.8 (3)
O14—N8—O13	124.6 (3)	C40—C41—H41	119.1
O14—N8—C37	117.2 (3)	N1—C1—H1	119.0
C29—N5—N6	109.0 (3)	N1—C1—C2	122.0 (3)
N1—C5—C6	114.0 (3)	C2—C1—H1	119.0
N1—C5—C4	122.9 (3)	N2—C10—C9	123.6 (3)
C4—C5—C6	123.1 (3)	N2—C10—H10	118.2
N4—C18—C17	115.4 (3)	C9—C10—H10	118.2
N4—C18—C19	121.8 (3)	C3—C2—H2	120.4
C19—C18—C17	122.8 (3)	C1—C2—C3	119.3 (3)
N2—C6—C5	114.8 (3)	C1—C2—H2	120.4
N2—C6—C7	122.4 (3)	C39—C40—C41	118.9 (3)
C7—C6—C5	122.7 (3)	C39—C40—H40	120.6
N3—C17—C18	114.9 (3)	C41—C40—H40	120.6
N3—C17—C16	122.8 (3)	O2—C12—H12A	109.5
C16—C17—C18	122.3 (3)	O2—C12—H12B	109.5
C18—C19—H19	120.2	O2—C12—H12C	109.5
C18—C19—C20	119.6 (3)	H12A—C12—H12B	109.5
C20—C19—H19	120.2	H12A—C12—H12C	109.5
O4—C20—C19	115.7 (3)	H12B—C12—H12C	109.5
O4—C20—C21	125.9 (3)	C33—C34—H34	120.6
C21—C20—C19	118.3 (3)	C33—C34—C35	118.8 (4)
C27—C28—N9	125.9 (3)	C35—C34—H34	120.6
C29—C28—N9	128.3 (3)	C30—C35—H35	118.6
C29—C28—C27	105.5 (3)	C34—C35—C30	122.8 (4)
C32—C31—N10	115.2 (3)	C34—C35—H35	118.6
C32—C31—C30	123.1 (3)	O3—C23—H23A	109.5
C30—C31—N10	121.5 (3)	O3—C23—H23B	109.5
C5—C4—H4	120.9	O3—C23—H23C	109.5
C5—C4—C3	118.2 (3)	H23A—C23—H23B	109.5
C3—C4—H4	120.9	H23A—C23—H23C	109.5
C31—C32—H32	121.0	H23B—C23—H23C	109.5
C31—C32—C33	118.0 (3)	O4—C24—H24A	109.5
C33—C32—H32	121.0	O4—C24—H24B	109.5
C37—C36—C27	126.2 (3)	O4—C24—H24C	109.5
C37—C36—C41	116.3 (3)	H24A—C24—H24B	109.5
C41—C36—C27	117.4 (3)	H24A—C24—H24C	109.5
N6—C27—C28	108.9 (3)	H24B—C24—H24C	109.5
N6—C27—C36	118.3 (3)	O1—C11—H11A	109.5
C28—C27—C36	132.8 (3)	O1—C11—H11B	109.5
O2—C8—C7	115.3 (3)	O1—C11—H11C	109.5
O2—C8—C9	125.8 (3)	H11A—C11—H11B	109.5
C9—C8—C7	118.8 (3)	H11A—C11—H11C	109.5
C6—C7—C8	118.9 (3)	H11B—C11—H11C	109.5
C6—C7—H7	120.5	C25—C26—H26A	109.5
C8—C7—H7	120.5	C25—C26—H26B	109.5
N5—C29—C28	108.3 (3)	C25—C26—H26C	109.5
N5—C29—C30	117.2 (3)	H26A—C26—H26B	109.5
C28—C29—C30	134.5 (3)	H26A—C26—H26C	109.5
O1—C3—C4	125.1 (3)	H26B—C26—H26C	109.5
O1—C3—C2	115.9 (3)	H17A—O17—H17B	104.5
C2—C3—C4	119.0 (3)		
			
Cu1—O5—C25—O6	−6.3 (6)	C5—C4—C3—C2	2.2 (6)
Cu1—O5—C25—C26	170.8 (3)	C18—N4—C22—C21	1.2 (5)
Cu1—N4—C18—C17	6.9 (4)	C18—C17—C16—C15	178.2 (3)
Cu1—N4—C18—C19	−171.4 (2)	C18—C19—C20—O4	179.5 (3)
Cu1—N4—C22—C21	171.9 (3)	C18—C19—C20—C21	0.1 (5)
Cu1—N1—C5—C6	−0.9 (4)	C6—N2—C10—C9	1.5 (6)
Cu1—N1—C5—C4	179.5 (3)	C6—C5—C4—C3	177.9 (3)
Cu1—N1—C1—C2	−177.6 (3)	C17—N3—C13—C14	0.8 (5)
Cu1—N2—C6—C5	−3.3 (4)	C17—C18—C19—C20	−178.6 (3)
Cu1—N2—C6—C7	176.9 (3)	C17—C16—C15—O3	−179.6 (3)
Cu1—N2—C10—C9	−175.6 (3)	C17—C16—C15—C14	0.4 (5)
Cu1—N3—C17—C18	5.7 (3)	C19—C18—C17—N3	169.9 (3)
Cu1—N3—C17—C16	−174.8 (3)	C19—C18—C17—C16	−9.6 (5)
Cu1—N3—C13—C14	175.3 (3)	C19—C20—C21—C22	0.9 (5)
O2—C8—C7—C6	−179.6 (3)	C20—C21—C22—N4	−1.6 (6)
O2—C8—C9—C10	−179.4 (4)	C28—C29—C30—C31	44.2 (7)
O4—C20—C21—C22	−178.4 (3)	C28—C29—C30—C35	−138.8 (5)
O11—N9—C28—C27	−168.9 (3)	C31—C32—C33—N11	−174.8 (3)
O11—N9—C28—C29	3.7 (6)	C31—C32—C33—C34	2.8 (6)
N4—C18—C17—N3	−8.3 (4)	C31—C30—C35—C34	0.8 (7)
N4—C18—C17—C16	172.2 (3)	C4—C5—C6—N2	−177.6 (3)
N4—C18—C19—C20	−0.5 (5)	C4—C5—C6—C7	2.2 (5)
O1—C3—C2—C1	179.6 (4)	C4—C3—C2—C1	−0.5 (6)
O10—N10—C31—C32	−141.1 (3)	C32—C31—C30—C29	177.2 (4)
O10—N10—C31—C30	33.6 (5)	C32—C31—C30—C35	0.2 (6)
N1—C5—C6—N2	2.8 (4)	C32—C33—C34—C35	−1.9 (7)
N1—C5—C6—C7	−177.4 (3)	C36—C37—C38—C39	−0.8 (6)
N1—C5—C4—C3	−2.5 (5)	C36—C41—C40—C39	−2.0 (6)
N1—C1—C2—C3	−1.1 (6)	C27—N6—N5—C29	−0.8 (4)
O3—C15—C14—C13	−179.0 (4)	C27—C28—C29—N5	0.4 (4)
N2—C6—C7—C8	−1.1 (5)	C27—C28—C29—C30	179.1 (4)
N3—C17—C16—C15	−1.3 (5)	C27—C36—C37—N8	0.3 (6)
N3—C13—C14—C15	−1.6 (6)	C27—C36—C37—C38	−177.9 (3)
O9—N10—C31—C32	36.0 (5)	C27—C36—C41—C40	179.6 (3)
O9—N10—C31—C30	−149.3 (4)	C8—C9—C10—N2	−0.6 (6)
O16—N7—C39—C38	152.5 (4)	C7—C8—C9—C10	−1.2 (6)
O16—N7—C39—C40	−28.0 (5)	C29—C28—C27—N6	−0.8 (4)
O12—N9—C28—C27	10.5 (6)	C29—C28—C27—C36	−178.3 (4)
O12—N9—C28—C29	−176.9 (4)	C29—C30—C35—C34	−176.5 (4)
N9—C28—C27—N6	173.2 (3)	C16—C15—C14—C13	1.0 (5)
N9—C28—C27—C36	−4.4 (6)	C37—C36—C27—N6	123.7 (4)
N9—C28—C29—N5	−173.4 (3)	C37—C36—C27—C28	−59.0 (6)
N9—C28—C29—C30	5.3 (7)	C37—C36—C41—C40	1.8 (5)
O8—N11—C33—C32	7.7 (6)	C13—N3—C17—C18	−178.8 (3)
O8—N11—C33—C34	−170.0 (4)	C13—N3—C17—C16	0.7 (5)
N10—C31—C32—C33	172.7 (3)	C30—C31—C32—C33	−2.0 (5)
N10—C31—C30—C29	2.9 (6)	C22—N4—C18—C17	178.1 (3)
N10—C31—C30—C35	−174.1 (4)	C22—N4—C18—C19	−0.1 (5)
O13—N8—C37—C36	−31.5 (5)	C33—C34—C35—C30	0.0 (8)
O13—N8—C37—C38	146.8 (4)	C9—C8—C7—C6	2.0 (5)
N11—C33—C34—C35	175.7 (4)	C38—C39—C40—C41	0.7 (6)
O7—N11—C33—C32	−172.4 (4)	C41—C36—C27—N6	−53.8 (5)
O7—N11—C33—C34	9.8 (6)	C41—C36—C27—C28	123.5 (4)
N6—N5—C29—C28	0.2 (4)	C41—C36—C37—N8	177.8 (3)
N6—N5—C29—C30	−178.8 (3)	C41—C36—C37—C38	−0.3 (5)
N7—C39—C38—C37	−179.9 (3)	C1—N1—C5—C6	−179.5 (3)
N7—C39—C40—C41	−178.7 (3)	C1—N1—C5—C4	0.9 (5)
N8—C37—C38—C39	−179.1 (3)	C10—N2—C6—C5	179.2 (3)
O15—N7—C39—C38	−26.5 (5)	C10—N2—C6—C7	−0.6 (5)
O15—N7—C39—C40	153.0 (4)	C40—C39—C38—C37	0.6 (6)
N5—N6—C27—C28	1.0 (4)	C12—O2—C8—C7	173.2 (3)
N5—N6—C27—C36	178.9 (3)	C12—O2—C8—C9	−8.5 (6)
N5—C29—C30—C31	−137.1 (4)	C23—O3—C15—C16	−177.7 (4)
N5—C29—C30—C35	39.9 (5)	C23—O3—C15—C14	2.3 (6)
O14—N8—C37—C36	149.8 (4)	C24—O4—C20—C19	176.1 (4)
O14—N8—C37—C38	−31.9 (5)	C24—O4—C20—C21	−4.5 (5)
C5—N1—C1—C2	0.9 (6)	C11—O1—C3—C4	−4.8 (6)
C5—C6—C7—C8	179.1 (3)	C11—O1—C3—C2	175.0 (4)
C5—C4—C3—O1	−177.9 (4)		

### (Acetato-κO)bis(4,4'-dimethoxy-2,2'-bipyridine-κ2N,N')copper(II) 3,5-bis(2,4-dinitrophenyl)-4-nitropyrazolate monohydrate Hydrogen-bond geometry (Å, º)

**Table:**

*D*—H···*A*	*D*—H	H···*A*	*D*···*A*	*D*—H···*A*
O17—H17*A*···N5	0.85	2.08	2.920 (7)	168
O17—H17*B*···O6^i^	0.85	2.40	3.168 (7)	151

Symmetry code: (i) −*x*+1, −*y*, −*z*+1.
